# Focal Epilepsy and the *Clock* Gene

**DOI:** 10.15844/pedneurbriefs-32-6

**Published:** 2018-09-05

**Authors:** Roberto Refinetti

**Affiliations:** 1 Department of Psychological Science, Boise State University, Boise, Idaho

**Keywords:** Focal Cortical Dysplasia, Human Epileptogenic Tissue, Clock-Flox Mouse Line

## Abstract

Investigators from Children’s National Medical Center, Wenzhou Medical University, Virginia Tech, University of Colorado, University of Virginia, Georgetown University, University of Maryland, and Brown University performed transcriptome analysis on human epileptogenic tissue and extended the investigation by creating and testing mouse lines with targeted genetic deletions of the *Clock* gene.

Investigators from Children’s National Medical Center, Wenzhou Medical University, Virginia Tech, University of Colorado, University of Virginia, Georgetown University, University of Maryland, and Brown University performed transcriptome analysis on human epileptogenic tissue and extended the investigation by creating and testing mouse lines with targeted genetic deletions of the *Clock* gene.

Because one of the genes that make up the molecular mechanism of the circadian clock (namely, *Bmal1*) had been previously shown to alter seizure threshold in mice, the authors of this article evaluated the expression of the *Clock* gene (*Bmal1*’s partner in the positive loop of the circadian clock) in brain tissue from patients with intractable epilepsy. They found decreased CLOCK protein in epileptogenic tissue as compared to control tissue. Next, they generated mouse lines with deletion of *Clock* in cortical excitatory neurons (*Emx-Cre;Clock^flox/flox^*) and found that these mice showed diminished seizure threshold and their excitatory neurons exhibited spontaneous epileptiform discharges. The authors also noticed that *Emx-Cre;Clock^flox/flox^* mice have defects in dendritic spines similar to spine defects seen in human epileptogenic tissue and that neurons from both the mutant mice and human tissue show decreased spontaneous inhibitory post-synaptic currents.

The authors conclude that the similarities between brain tissue from *Emx-Cre;Clock^flox/flox^* mice and from epileptic patients suggest that disruption of CLOCK may be a central component of dysfunctional cortical circuits involved in the generation of focal epilepsy. [1]

COMMENTARY. Because focal cortical dysplasia is the most common cause of intractable focal epilepsy in children [2], the findings of this study are clearly of interest to pediatric neurologists. How soon these research findings will lead to effective treatments, however, will depend on follow-up studies. *Clock* gene variants have been associated with chronotype variations [3], sleep disorders [4], obesity [5], alcoholism [6], and various other disorders, and it remains to be determined how specific the disruption of *Clock* expression is for the generation of focal epilepsy. The situation is further complicated by the fact that, at least in some areas of the brain, the circadian clock can remain operational using *Npas2* instead of *Clock* when *Clock* is knocked out [7].

If the loss of *Clock* transcriptional activity is specific for the generation of focal epilepsy, its mechanism of action must be further investigated. The increased excitability of pyramidal neurons may be due to the loss of inhibition on principal neurons, and the authors recognize that further research is necessary to clarify how the loss of *Clock*-mediated transcription in excitatory neurons results in impaired inhibitory activity in their presynaptic partners. They plan to investigate whether the primary result of CLOCK loss of function is a synaptic change or a circuit change (or both).

## Disclosures

The author(s) have declared that no competing interests exist.

## References

[1] Li P, Fu X, Smith NA, Ziobro J, Curiel J, Tenga MJ (2017). Loss of CLOCK results in dysfunction of brain circuits underlying focal epilepsy. Neuron.

[2] Lerner JT, Salamon N, Hauptman JS, Velasco TR, Hemb M, Wu JY (2009). Assessment and surgical outcomes for mild type I and severe type II cortical dysplasia: a critical review and the UCLA experience. Epilepsia.

[3] Katzenberg D, Young T, Finn L, Lin L, King DP, Takahashi JS (1998). A CLOCK polymorphism associated with human diurnal preference. Sleep.

[4] Iwase T, Kajimura N, Uchiyama M, Ebisawa T, Yoshimura K, Kamei Y (2002). Mutation screening of the human *Clock* gene in circadian rhythm sleep disorders. Psychiatry Res.

[5] Garaulet M, Corbalán MD, Madrid JA, Morales E, Baraza JC, Lee YC (2010). *CLOCK* gene is implicated in weight reduction in obese patients participating in a dietary programme based on the Mediterranean diet. Int J Obes.

[6] Sjöholm LK, Kovanen L, Saarikoski ST, Schalling M, Lavebratt C, Partonen T (2010). CLOCK is suggested to associate with comorbid alcohol use and depressive disorders. J Circadian Rhythms.

[7] Debruyne JP, Noton E, Lambert CM, Maywood ES, Weaver DR, Reppert SM (2006). A clock shock: mouse CLOCK is not required for circadian oscillator function. Neuron.

